# A Cost-benefit Analysis of Using Polyethylene Glycol Hydrogel Sealant versus Fibrin Glue as a Dural Sealant for Posterior Fossa Surgery in the United States

**DOI:** 10.36469/001c.35589

**Published:** 2017-08-09

**Authors:** Marissa J. Carter

**Affiliations:** 1 Strategic Solutions, Inc.

**Keywords:** posterior fossa neurosurgery, dural sealant, DuraSeal, fibrin glue, cost-benefit, CSF leak, health economics

## Abstract

**Background:** Cerebrospinal fluid (CSF) leaks are a well-known complication of posterior fossa neurosurgery. The use of dural repair adjuncts has been associated with fewer CSF leaks and subsequently, lower medical costs.

**Objectives:** To determine if the use of polyethylene glycol hydrogel sealant (DuraSeal dural sealant system [DuraSeal]), resulted in cost savings in the United States compared to use of fibrin glue in posterior fossa neurosurgery based on a published study of these two products.

**Methods:** Primary analysis was based on a published cohort study (100 patients per cohort). Adjustments were made to account for the longer follow-up time of the fibrin glue cohort to better capture the incidence of CSF leaks, pseudomeningocele, wound infection, and meningitis using additional data from the literature. Resource utilization was calculated from literature studies and confirmed with consultations from two practicing neurosurgeons. The effective time horizon was 19 months. Undiscounted Medicare payments were used to calculate unit costs from a payer’s perspective based on hospital stay, cost of sealant application, and cost of complications. One-way sensitivity analysis was used to examine changes in costs as a result of model input changes.

**Results:** In the base case, the cost of the hospital stay for the original surgery, which excludes the cost of complications, dominated costs for both cohorts. On a per patient basis, the use of fibrin glue cost $1666 more than the use of DuraSeal. Sensitivity analysis showed that using lumbar drainage instead of operative repair for a CSF leak reduced cost savings to $680. Holding the incidence of CSF leaks constant for the fibrin glue cohort while increasing the incidence for the DuraSeal cohort by 50% or 300% resulted in cost savings of $1438 and $755, respectively.

**Conclusions:** The results of this study demonstrate a positive, consistent cost-benefit of DuraSeal compared to fibrin glue based on a cohort study of real world patients who underwent posterior fossa neurosurgery.

## Background

Cerebrospinal fluid (CSF) leaks are a well-known complication of posterior fossa surgery. Although authors of the largest observational study conducted to date found a CSF leak prevalence of 13% based on 15-25 year old data in posterior fossa surgeries[1] approaches and techniques have not changed sufficiently enough to substantially lower this rate. Most CSF leaks tend to occur in the early postoperative period.[1-4] Neurosurgeons have used a wide variety of products or procedures to prevent CSF leaks from occurring following cranial surgery with varying degrees of success. These include the 'sandwich' technique[5]; autologous grafts, such as fat or pericranium[6,7]; dural grafts, such as collagen matrices (DuraGen®)[8-10]; fibrin glue[11]; fibrin sealant patches (TachoSil®)[12]; and polyethylene glycol hydrogel dural sealants (DuraSeal®) (DS).[13]

There is some evidence from the literature that use of dural repair adjuncts can result in fewer complications, and consequently lower monetary costs. For example, Kassam et al[14] reported that a retrospective cohort of 72 patients who underwent neurosurgical procedures and concurrent use of fibrin glue experienced no CSF leaks, but a historical cohort of similarly treated 181 patients who had no fibrin glue experienced 16 CSF leaks at an estimated aggregate cost of $165 651. Likewise, Kus et al[15] indicated that DS use may reduce overall yearly hospital costs compared to using fibrin glue in endoscopic pituitary surgery. Finally, Grotenhuis[16] reported in a single institution, retrospective Dutch study comparing 412 patients who had undergone neurosurgical procedures during 1999 with a smaller group of 46 patients in whom prophylactic use of DS had been used could save €550 per patient in 2004 costs.

All of these studies have significant limitations. In particular, both more generalizable costs and sensitivity analysis are needed to better understand if cost savings can be consistently obtained using dural sealants. Another challenge is the choice of comparator. So far, there is no standard method for categorizing and/or repairing CSF leaks in neurosurgery; rather a mélange of techniques and materials are employed.

To better understand if polyethylene glycol hydrogel dural sealant can provide consistent cost savings when used prophylactically for dural closure following posterior fossa surgery, a cost-benefit study was undertaken. The study used the results presented by Than et al,[17] who investigated the outcomes of a prospective cohort treated with DuraSeal, an FDA–approved sealant used as an adjunct to sutured dural repair during cranial surgery. This cohort was matched to a historical cohort that had been treated with fibrin glue (FG) used off-label as an adjunct for dural repair. Study results are reported in terms of (a) whether the use of DS resulted in cost savings, (b) under what conditions were cost savings obtained, and (c) the magnitude of cost savings when key variables were subject to sensitivity analysis.

## Methods

### <u>*Clinical Outcomes*</u>

#### *Description of Study Cohort*

Than et al[17] compared the clinical outcomes of a retrospective cohort of patients who underwent dural closure augmentation with FG (n=100) to a prospective cohort augmented with DS (n=100). All patients underwent posterior fossa surgery by craniotomy or craniectomy. The surgeries were performed in a single institution between September 2004 and April 2006 by the same surgeons in both groups.

*Incidence of Complications for DS Cohort*

In general, if the mean time to a complication following neurosurgery is significantly less in one cohort compared to another, then a shorter follow-up time in the faster time-to-complication cohort may be reasonable in terms of analysis compared to slower time-to-complication cohort. However, this assumption can be strongly influenced by the incidence of the complication; for example, the situation may also need to take into account the mean incidence of complications: a larger incidence of complications and their sequiturs may take a longer time to resolve. Although the mean time to a CSF leak was reported as 9.1 (SD [standard deviation]: 5.3) and 28 (SD: 14) days for the FG and DS cohorts, respectively, and the complication rate was higher for the FG cohort (22% vs 16% for the DS cohort),[17] the mean follow-up time of the FG cohort was much longer than the DS cohort (19 months vs 2.9 months). Consequently, adjustments need to be made for the base case for the 4 reported complications (CSF leak, pseudomeningocele, wound infection, and meningitis) to fully capture their incidence in the DS cohort.

Time to a CSF leak is less likely to follow a normal curve (Gaussian) function and more likely to follow a positive gamma function in which there is a short rise to a maximum followed by a long tail (i.e., many acute post-surgical complications tend to happen sooner than later[2]). Using an applet to plot the distribution function[18] and setting α to 3, different values of β were imputed until the resultant mean and SD (mean: 27.3; SD: 15.75) approximated reported values (mean: 28; SD: 14) with β set to 0.11. With negligible probability of time to a CSF leak event set to 0.01, the time at which this occurs based on the probability function was determined to be approximately 75 days (Figure 1). Although no detailed time to CSF leaks could be found for posterior fossa surgery in the literature, more detailed information is available for trans-sphenoidal surgery. While the approaches between these surgeries are different, the concept, supported by study data, is that CSF leaks happen in the early-postoperative period while tissues are still healing after surgery.[1,4] Han et al[3] reported that 73% of post-operative CSF leaks occurred within 25 days and 92% within 50 days whereas Naunheim et al[2] reported that all CSFs leaks occurred within 12 days. Thus, the data do suggest that the gamma function is a reasonable approximation to use in describing time to CSF leak.

**Figure 1. Approximating Time to CSF Leak in the DS Cohort**

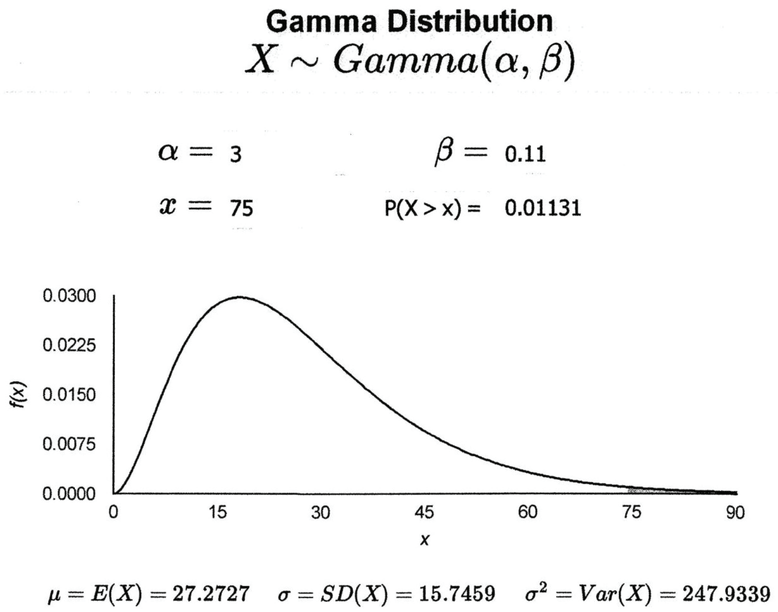

A positive gamma function was used to approximate the time to CSF leak (α=3, β=0.11, negligible probability~0.01).
CSF: Cerebrospinal fluid; DS: DuraSeal

While the gamma function solved the problem of likely time to CSF leak, some patients may have had too short of a follow-up period based on a mean follow up time of 2.9 months. A Gaussian function was next constructed to approximate the follow-up time for the DS cohort using a mean of 88 days (~2.9 months)[17] and setting the SD to 30, which approximates the left tail of the distribution function to about 0 days. The area under the curve from 0 to 75 days is approximately 0.33, which means that theoretically about one third of patients may have had too short of a follow-up time to determine whether a CSF leak occurred. However, in a proportion of these patients there would have been adequate time to detect a CSF leak if they were in the hospital after the initial surgery for a long enough period of time. Mean length of stay (LOS) including surgery for the Than[17] study was 5 days (range: 1-22); other studies reported 3.8 days (range: 2-8)[6] and 11.8 (range: 3-120),[19] indicating that LOS in the Than study was not unusual. Using a gamma function to approximate LOS for the Than study[17] (P~0.01 for 22 days), the resultant curve ($\alpha$=1.15, $\beta$=0.22) showed that approximately 60% of patients would have had an initial stay of 5 days or less.

In regard to time to CSF leak, based on the gamma function used for time to CSF leak, this equates to about 2% of patients probably having too short of a time for CSF leak detection if any follow-up was not recorded. Since we do not know the true proportion of patients that had too short of a time for CSF leak detection based on follow-up time data reported by Than et al,[17] with probable extremes of 2% and 33%, a figure of 20% was chosen to be conservative. Thus, the incidence of CSF leaks for the DS cohort was set to 0.024 (0.02 x 1.2), in which 0.02 represents the incidence rate reported by Than et al.[17]

Because the development of pseudomeningoceles would be expected to parallel that of CSF leaks based on the same physiological mechanism,[20] adjustment of the incidence of this complication used the same factor as for CSF leaks: 0.096 (0.08 x 1.2).

Dashti et al[21] observed only about 0.5% of patients develop a deep infection following cranial surgery that requires reoperation (median time to event: 1.5 months). As there is no a priori reason to assume that deep infections rates are different between the cohorts, costs of treating these infections were not included. Given that superficial infections are simply treated and occur relatively quickly—for example, Naunheim et al[2] reported that all infections occurred within 15 days—the incidence of these complications was not adjusted in the DS cohort.

Meningitis complications following posterior fossa surgery are typically of aseptic etiology.[21] In addition, there is a strong association of aseptic meningitis with CSF leakage,[22] with complications occurring within 3 weeks post-surgery.[23] Consequently, adjustment of the incidence of this complication used the same factor as for CSF leaks: 0.024 (0.02 x 1.2).

The adjusted incidence rates for the four complications in the DS cohort along with the incidence rates in the FG cohort are shown in Table 1. Note that adjustments were made only to the DS cohort to account for its shorter follow-up time compared to the FG cohort. The complication rates for the FG cohort, on the other hand, were unchanged from those reported in the Than[17] study.

*Treatment of Complications*

CSF leaks are incisional; based on the literature[1,17,24] and consultations with 2 neurosurgeons in the United States, it was decided that in the base case, all patients should have operative repair in-hospital (5 days in hospital).

A search of the literature suggested that 20% of patients would require clinical intervention for pseudomeningoceles.[9,19] Consultations with 2 US neurosurgeons indicated that 50% of the time one would treat pseudomeningoceles with operative repair in hospital, and 50% of the time treatment would comprise lumbar puncture and/or drainage in hospital. Consequently, 20% of the pseudomeningoceles reported in each cohort were assigned clinical intervention, with half having operative repair in hospital and half having a lumbar puncture in hospital (both requiring 5 days in hospital).

The treatment for superficial surgical site infection is a short course of antibiotics. Amoxicillin (500 mg, twice daily) for 1 week was chosen as the antibiotic treatment with 2 follow-up outpatient visits assigned to each patient in addition to determine whether the infection was resolved.

Based on a review of the literature in regard to aseptic meningitis,[1,9,25] 75% of cases were assigned a 2-week course of dexamethasone (2 x 6 mg daily), and 25% assigned the same regimen for 4 months. In addition, based on consultations with 2 US neurosurgeons, it was decided that 50% of patients would get a lumbar puncture in the OR plus a CSF culture and antibiotics for 1 week, with 2 days in hospital and 1 post-hospital outpatient visit. The other 50% of patients were assigned 2 outpatient visits with no lumbar puncture.

**Table 1. Incidence and Adjusted Incidence of the 4 Complications in the FG and DS Cohorts, Respectively**

| Complication | Incidence* | |
|---|---|---|
| | FG Cohort | DS Cohort |
| CSF leak | 0.1 | 0.024 |
| Pseudomeningocele | 0.05 | 0.096 |
| Wound infection | 0.02 | 0.04 |
| Aseptic meningitis | 0.05 | 0.024 |

CSF: Cerebrospinal fluid; FG: fibrin glue; DS: DuraSeal

*Based on a follow-up of approximately 19 months in both cohorts

*Economic Analysis*

The cost-benefit analysis captured the costs of 4 post-operative complications for the 2 cohorts.[17] Tisseel (frozen; Baxter Healthcare Corp., Deerfield, IL) was selected as the FG material and DuraSeal (dural sealant; Integra Life Sciences, Plainsboro, NJ) was selected as the DS material. Based on the mean follow-up time of the FG cohort, the time horizon was approximately 19 months. Costs were undiscounted because the majority of costs occurred in the first year.

*Unit Costs*

The perspective was that of the healthcare payer in the United States. All costs were based on 2016 Medicare national average payment rates with the exception of DS and FG materials and drug costs. DS and FG sealant costs were obtained from an IMS Health (Danbury, CT) Hospital Supply Index audit as the average selling price (ASP) based on moving annual total (MAT) sales and units through the second quarter of 2016, whereas the costs of drugs were obtained from average prices at drugs.com.[26]

Based on an analysis of the MAT data (from 350 hospitals for all application of these products), the ASP for FG for the base case was calculated using a 3:1 ratio of frozen 10 mL to 4 mL.

For 2013, charges per hospital inpatient day (ie, approximate per diem rate) were estimated at $2157,[27] which is $2235 in 2016 prices.[28] The Medicare PCR (payment to charge ratio) reported by Smith et al[29] was

0.32 in 2006 which equates to a Medicare payment of $715 per diem for a hospital stay in 2016 assuming no PCR changes.

Physician and facility fees for each relevant CPT code under the Outpatient Prospective Payment System (OPPs) system were obtained from the Centers for Medicare and Medicaid Services (CMS).[30,31] Fees for codes 87070 and 87205, were obtained from CMS using the 57 states and states' regions' clinical diagnostic laboratory fees to determine the national average cost.[32] The fee for inpatient CPT code 62272 was estimated using the OPPS system,[30] with a weighting factor of 3.6 applied based on General Accounting Office (GAO) findings.[33] The mean Medicare payment for CPT code 61678 was estimated using DRG 027 (craniotomy and endovascular intracranial procedures without complications or major complications), based on 2014 Medicare inpatient charge and cost data.[34]

Summary unit costs and codes are shown in Table 2.

**Table 2. Unit Cost Table**

| Item | Description (Unit) | CPT/ HCPCS Code | Medicare 2016 Allowable Charges ($) | Source |
|---|---|---|---|---|
| Hospital stay | Per diem rate, no special hospital unit (1 day) | N/A | 715 | Kaiser Family Foundation, with adjustments based on data from Smith et al[29] |
| Outpatient visit | Clinic visit (visit) | G0463 | 102.12 | CMS |
| DuraSeal | Cranial version, 5mL (1 application set) | N/A | 835.77 | IMS Health |
| Tisseel | Frozen, 4mL (1 application set) | N/A | 388.08 | IMS Health |
| Operative repair | Repair and/or reconstruction of surgical defects of skull base procedures | 61618 | DRG 027: 15 397 | CMS |
| Lumbar puncture | Spinal puncture, therapeutic, for drainage of cerebrospinal fluid, by needle or catheter. | 62272 | Physician: 311.94* Facility: 2106.61* | CMS with weighting from General Accounting Office[33] |
| CSF culture | Culture, any other source except urine, blood or stool, aerobic, with isolation and presumptive identification of isolates | 87070 | 11.74 | CMS |
| | Smear, primary source with interpretation; Gram or Giemsa stain for bacteria, fungi or cell types | 87205 | 5.82 | |
| Steroids | Dexamethasone (6 mg, 2-week course) | N/A | 4.00 | drugs.com |
| Antibiotics | Amoxicillin tablets (500 mg, twice daily, 1-week course) | N/A | 27.40 | drugs.com |

N/A: not applicable; *Weighting factor applied.
Costs are national averages of Medicare allowable charges for 2016, except for the sealant products and drugs.

CMS: Centers for Medicare and Medicaid Services; CPT: current procedural terminology; DRG: diagnosis related group; HCPCS: Health Care Procedural Coding System

*Calculation of Cohort Costs*

To calculate the difference in costs between cohorts, the following element costs were calculated: hospital stay, cost of sealant application, and cost of complications. It was assumed that costs of procedures other than sealant application would be the same for both cohorts in the initial hospital stay. The costs of hospital stays for complications are included in each complication where applicable.

Than et al[17] reported a mean hospital stay of 5.0 and 6.4 days for the DS and FG cohorts, respectively. It was assumed that these figures also included 10 days total for the two patients in the DS cohort who were readmitted to hospital for a CSF leak, and similarly, 47 days for the 10 patients in the FG cohort. To calculate the initial cost of hospital stay for the DS and FG cohorts, excluding the days in hospital required for complications, the following calculations were performed:

$$HS_{DS} = ((5 \text{ x } 100) – 10) \text{ x } \$715$$
$$HS_{FG} = (6.4 \text{ x } 100 – 47) \text{ x } \$715$$

where $HS_{DS}$ is the total cost for hospital stay for the DS cohort prior to complications, and $HS_{FG}$ is the same metric for the FG cohort.

Costs of complications were based on numbers of patients in each cohort experiencing the complications reported in the Than study,[17] the cost of the sealant, and the adjustment factors described in treatment of complications section, using the unit costs listed in Table 2.

*Sensitivity Analysis*

A change in the mix of cases in regard to neurological diagnosis and treatment, as well as different practice patterns are likely to affect assumptions used in the base case. The variables selected in the one-way sensitivity analysis and their chosen values reflect reasonable and conservative estimates (Table 3). For example, the base case assumes 100% operative repair to manage CSF leaks while the sensitivity analysis calculates the costs of 100% lumbar drainage, which permits cost estimates of different approach mixes to managing CSF leaks. In the lumbar drainage scenario it is assumed that the hospital stay is 5 days.[23] One additional 3-way and one additional 2-way sensitivity analysis were undertaken to test scenarios in which (a) differences between institutions might occur (DS cost was increased by 25%, the FG sealant used a 1:1 mix of 10mL and 4mL frozen Tisseel, and lumbar drainage was used instead of operative repair to address CSF leaks); and (b) clinical outcomes are different (the incidence of CSF leaks and pseudomeningoceles was increased by 50% in the DS cohort while simultaneously decreasing the incidence of these complications in the FG cohort by 50%). Sensitivity analysis of treatments for aseptic meningitis and wound infection as well as CPT code 62272 costs was not undertaken because the overall changes in costs between the cohorts were much smaller in relation to other variables.

**Table 3. Variables Used in Sensitivity Analysis with Rationale for Using Them and Values Used in the Analysis.**

| Variable | Rationale | Basis | Values |
|---|---|---|---|
| (1) Mean days in hospital | Although there is a mean differential in days between DS and FG cohorts in the Than study[12] differential could be less or more. | Change mean differential number of days in hospital before complications | ±50% |
| (2) Cost of sealant | Cost of sealants depends in part on volume used for the Tisseel kit (ie, 4mL or 10 mL) as well as average selling price. | (a) Change price by percentage for each sealant<br><br>(b) Change base case volume ratio for Tisseel while keeping DS costs the same | ±25%<br><br>(a) 10 mL: 90%, 4 mL: 10%<br><br>(b) 10 mL: 50%, 4 mL: 50% |
| (3) CSF leak | The incidence of CSF leaks is likely to vary in regard to the mix of neurological surgery as well as local practice even using the two different sealant products. | (a) Change incidence of one cohort while holding the incidence of the other cohort constant<br><br>(b) Assume that lumbar drainage is exclusive | ±50%; 3-fold increase in rate for DS<br><br>Lumbar drain 100% |
| (4) Pseudomeningocele | The incidence of pseudomeningoceles needing clinical intervention is likely to vary in regard to the mix of neurological surgery as well as local practice even using the two different sealant products. | Change incidence of one cohort while holding the incidence of the other cohort constant | ±50% |
| (5) Cost of procedures | The estimated cost for CPT code 61618 is likely to be an overestimate for Medicare and an underestimate for private insurance. | Change unit costs | ±25% |

CSF: Cerebrospinal fluid; DS: DuraSeal; FG: fibrin glue

Note: if the incidence was 0.02 then a 50% increase would mean 0.03 and a -50% decrease would mean 0.01.

## Results

### *Base Case*

In the base case, the cost of the hospital stay for the original surgery, which excludes the cost of complications, dominated costs for both cohorts, constituting 63-69% of total cost (DS cohort: $350 350; FG cohort: $423 995; Table 4). While the costs of FG application were about 46% of the DS application (DS cohort: $83 577; FG cohort: $38 807), the cost of CSF leaks was over 4-fold more for the FG cohort compared to the DS cohort (DS cohort: $45 533; FG cohort: $189 720). Other complications, including pseudomeningoceles, aseptic meningitis, and wound infection were a much smaller percentage of total costs. On a per patient basis, the use of FG cost $1666 more than the use of DS.

**Table 4. Cost of Hospital Stay, Applied Sealant, and Complications**

| Component | Cost ($) | |
|---|---|---|
| | DS Cohort | FG Cohort |
| Hospital stay* | 350 350 | 423 995 |
| Sealant | 83 577 | 38 807 |
| CSF leak | 45 533 | 189 720 |
| Pseudomeningocele | 23 967 | 12 483 |
| Aseptic meningitis | 5066 | 10 555 |
| Wound infection | 927 | 463 |
| TOTAL | 509 420 | 676 022 |

CSF: Cerebrospinal fluid; DS: DuraSeal; FG: fibrin glue
*Before any complications

*Sensitivity Analysis*

Changes in costs for each cohort based on each of the one-way sensitivity analyses are shown in Table 5. The parameters/variables chosen were each varied from the base case scenario while keeping the values of the other parameters constant and the resulting medical costs were calculated for each cohort. For example, increasing the CSF leak incidence by 50% in the DS cohort while keeping the incidence the same (as the base case) in the FG cohort, the cost in the DS cohort increased from $45 533 (base case) to $83 577, compared to $189 720 for the FG cohort. Lowering the incidence of pseudomeningoceles for the FG cohort by 50% while keeping the same incidence for the DS cohort resulted in a lower cost of treatment of $6241 compared to the base case of $12 483. Likewise, when the ratio of FG sealant was changed to a 50% mix of 10mL and 4mL the cost was reduced to $32 495 from $38 807.

The changes on a per patient basis are shown in Figure 2. The vertical reference line represents the base case in which a patient treated with FG cost $1666 more than a patient treated with DS. As an example, the largest changes were observed when lumbar drainage was used instead of operative repair for CSF leaks while still having the same length of hospital stay. In this example the cost difference is narrowed because lumbar drainage is cheaper, resulting in a difference of only $680 on a per patient basis (blue horizontal bar), albeit still with less costs for DS (ie, FG is $680 more expensive than DS; Figure 2). The situation in which CSF leaks were managed 50% by operative repair and 50% by lumbar drainage equated to a cost savings of $1173 (data not shown in figure). When the incidence of CSF leaks was increased by 50% for the DS cohort, representing a rate of 3.6 cases per 100, while keeping the incidence for the FG cohort constant, the difference in costs on a per patient basis was $1438—still with less cost for a patient treated with DS (Figure 2). However, when the incidence for the DS cohort was decreased by the same amount while keeping that of the FG cohort constant, the difference in costs increased to $2044 (horizontal green bar). If the incidence of CSF leaks was tripled (7.2 cases per 100) in the DS cohort while holding the incidence of CSF leaks constant for the FG cohort, costs savings of $755 still resulted. Reducing the incidence of CSF leaks by 50% in the FG cohort while keeping the same incidence in the DS cohort as in the base case still resulted in cost savings of $717 per patient. Mean days in hospital also had a substantial influence with a difference between cohorts on a per patient basis of $1166 to $2167 and FG costs always more expensive. When the cost of the operative procedure was changed by ±25%, cost savings of $1391-1941 still accrued, about 17% of the base case on a dollar basis (Figure 2).

Other variables had far less influence. Changing the cost of sealant by ±25% resulted in cost savings of $1554-1778 per patient (doubling the cost of DS sealant alone resulted in costs savings of $830 in favor of DS treatment) and changing the volume ratio for FG (10 mL 50% or 90%) equated to cost savings of $1603-1704, both in favor of DS, but the change on a percentage basis was small, about 3-7% of the base case. Changing the incidence of pseudomeningoceles in the DS cohort by ±50% had a similar cost savings range ($1546-1786), and change on a percentage basis of the base case (7%).

The 2-way and 3-way sensitivity analyses represented a test of model assumptions in which practice or clinical outcomes differ at various institutions. In the 3-way analysis (DS cost increased by 25%, FG sealant was a 1:1 mix of 10mL and 4mL frozen Tisseel, and lumbar drainage was used to address CSF leaks) the cost difference on a per patient basis was $407 in favor of DS treatment. In the 2-way analysis (incidence of CSF leaks and pseudomeningoceles increased by 50% in the DS cohort and decreased 50% in the FG cohort) the cost difference was $308, still in favor of DS treatment.

**Table 5. One Way Sensitivity Analysis: Changes in Cohort Portion Costs for Each Variable Obtained by Changing Values of Key Variables**

| Variable | FG Cohort Portion Cost ($) | DS Cohort Portion Cost ($) |
|---|---|---|
| (1) Mean days in hospital | | |
| (a) +50% | 652 795 | 529 100 |
| (b) -50% | 195 195 | 171 600 |
| (2) Cost of sealant | | |
| (a) Change in price, +25% | 48 509 | 104 471 |
| (b) Change in price, -25% | 29 105 | 62 683 |
| (c) FG volume ratio, 10 mL: 90%, 4 mL: 10% | 42 594 | 83 577 |
| (d) FG volume ratio, 10 mL: 50%, 4 mL: 50% | 32 495 | 83 577 |
| (3) CSF leak | | |
| (a) DS: no change; FG: +50% | 284 580 | 45 533 |
| (b) DS: no change, FG: -50% | 94 860 | 45 533 |
| (c) DS: +50%; FG: no change | 189 720 | 83 577 |
| (d) DS: -50%; FG: no change | 189 720 | 16 539 |
| (e) All lumbar drainage | 59 936 | 14 385 |
| (4) Pseudomeningocele treatment | | |
| (a) DS: no change; FG: +50% | 18 724 | 23 967 |
| (b) DS: no change, FG: -50% | 6241 | 23 967 |
| (c) DS: +50%; FG: no change | 12 483 | 35 950 |
| (d) DS: -50%; FG: no change | 12 483 | 11 983 |
| (5) Cost of CPT 61618 procedure | | |
| (a) CSF leak & pseudomeningocele, +25% | 242 617 | 82 432 |
| (b) CSF leak & pseudomeningocele, –25% | 161 788 | 56 567 |

FG: fibrin glue; DS: DuraSeal; CSF: Cerebrospinal fluid

**Figure 2. Tornado Diagram Showing Expected Changes in Cost ($) from the One-way Sensitivity Analysis**

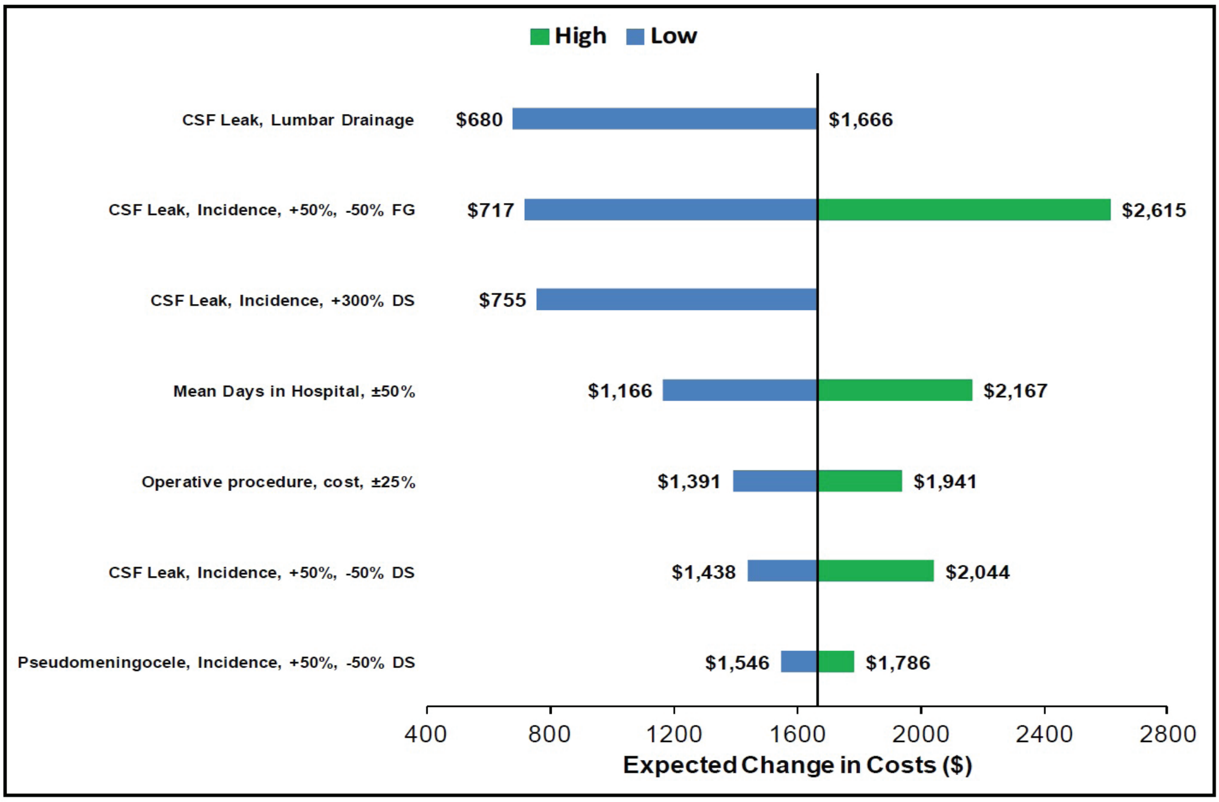

CSF: Cerebrospinal fluid

Results are expressed as low and high costs on a per patient basis, reflecting the smallest and largest differences between cohort costs for each analysis.

## Discussion

The results of the study demonstrate that, based on 2 real-world cohorts of patients who underwent posterior fossa neurosurgery, the use of DuraSeal consistently results in cost savings when compared to using fibrin glue as a dural sealant. Given that these costs are based on Medicare reimbursement payments, cost savings for private insurers are likely to be much higher. This is the first study to assess the cost-benefit of DuraSeal compared to fibrin glue in posterior fossa surgery.

A major criticism of the Than study is that follow-up times were too short for the DS cohort thereby not capturing the full incidence of complications compared to the FG cohort.[17] The methodology used in the base case and the one-way sensitivity analyses were specifically developed to adjust for this problem. Furthermore, even using the base case adjustment and a 3-fold increase in the incidence of CSF leaks, cost savings per patient still accrued in favor of DS use.

Another criticism leveled at the study is that DS did not significantly improve the rate of CSF leak and pseudomeningocele complications taken together.[17] This argument may be specious because it presumes that all pseudomeningoceles that develop require clinical intervention as opposed to conservative care. The limited data available from the literature[9,10,19] suggest that perhaps one fifth of pseudomeningoceles require clinical intervention, which is the proportion adopted for the base case. One-way sensitivity analyses also showed that even if the incidence rates are substantially changed—equivalent to increasing the proportion of pseudomeningoceles that require clinical intervention—substantial cost savings were still realized when DS is used instead of FG.

The magnitude of the cost savings for Medicare per patient, nearly $1700 in 2016 figures, represents only the quantifiable costs. While societal perspectives are deemed more encompassing of costs, it is far more common in health economics to use a payer perspective that reflects a national situation, such as Medicare in the United States. Although "hospital" perspectives are certainly of interest to administrators, the problem is that hospital charges and costs to provide services vary enormously both individually and regionally and thus it would be hard to generalize the difference between those costs and what hospitals receive from payers to determine what kind of savings hospitals could expect from using DS in posterior fossa neurosurgery.

It was not possible to assess complete hospitalization costs besides *per diem* charges because of insufficient information. Although it is possible that the DS and FG cohorts could have incurred substantially different charges compared to one another, the most important issue would be evaluating which charges were related to the original neurosurgery operation and complications versus charges that might be related to other comorbidities or medical conditions. Indeed, many health economics studies that explore hospitalization are faced with this same issue. There are also other practical problems that this study did not address, including the fact that the frozen FG requires ordering in advance and usage within 24 hours. Such problems are difficult to assess economically.

The results from this study are only applicable to the United States and not generalizable to other countries without adjustments of costs and procedures adapted to local practice. Moreover, given that so many different products and procedures are used more frequently in other countries it may be more appropriate to use other product comparators for cases in which a suitable and well-conducted study can be found as a basis for outcomes. This simply reflects the large diversity of products and approaches and preferences used in different regions of the world.

Strengths of this study include adjustments for unequal follow-up periods based on clinical outcomes from a real world cohort study, the use of Medicare payments as opposed to hospital charges, and a robust sensitivity analysis. There are also some limitations to this study. First, it was assumed that in general, complications would occur after the initial hospital stay for fibrin glue patients, which would not be true for a small proportion of patients in particular scenarios. Second, hospital stay costs were kept simple and did not include time spent in an ICU or other specialty unit for patients. Third, other complications may arise from posterior fossa neurosurgery and these costs were not included because they would be inestimable based on the results of the cohort study (ie, they did not occur or were not reported in the Than study[17]). An example of this would be the failure of lumbar drainage or operative repair to manage the leak, with further interventions needed to resolve the problem. Fourth, the generalizability of the results in this study is limited to a typical "mix" of surgeries; specific types of surgeries and their complications may have different costs with the result that the cost-benefit of using DS may depend in part on the type of surgery employed. Finally, it is possible that in some scenarios the assumptions used in this analysis will be in error because either practice patterns or clinical outcomes are different between institutions. Both these issues were explored in two separate 3-way and 2-way sensitivity analyses and both returned results in favor of DuraSeal treatment, although the costs savings were very much reduced on a per patient basis.

## Conclusions

CSF leaks are a known complication of posterior fossa surgery and incur significant medical costs. This study demonstrated a positive, consistent, cost-benefit of DuraSeal compared to fibrin glue based on a cohort study of real-word patients who underwent posterior fossa surgery. These findings could have meaningful implications for the management of dural repair during posterior fossa neurosurgery.

**Declaration of Funding**

This study and preparation of the manuscript was financially supported by Integra LifeSciences Corporation. Integra LifeSciences Corporation was not involved in developing the study design, data collection, data analysis, and interpretation but was involved in the decision to publish these results.

**Declaration of Financial/Other Relationships**

MJC is a consultant and was paid by Integra LifeSciences for this investigation.

**Author Contributions**

MJC conceived and designed the study, gathered, analyzed, and interpreted the data, drafted the paper and revised it critically for intellectual content, approved the final version to be published, and is accountable for all aspects of the work.

**Acknowledgments**

The assistance of Drs Michael Seiff (The Spine and Brain Institute) and Gerald Grant (Stanford University Medical Center) is gratefully acknowledged in regard to assistance with determining neurosurgical practice in the USA.
